# Immunogenicity in Rabbits of HIV-1 SOSIP Trimers from Clades A, B, and C, Given Individually, Sequentially, or in Combination

**DOI:** 10.1128/JVI.01957-17

**Published:** 2018-03-28

**Authors:** Alba Torrents de la Peña, Steven W. de Taeye, Kwinten Sliepen, Celia C. LaBranche, Judith A. Burger, Edith E. Schermer, David C. Montefiori, John P. Moore, Per Johan Klasse, Rogier W. Sanders

**Affiliations:** a Department of Medical Microbiology, Academic Medical Center, University of Amsterdam, Amsterdam, The Netherlands; b Department of Surgery, Duke University Medical Center, Durham, North Carolina, USA; c Department of Microbiology and Immunology, Weill Medical College of Cornell University, New York, New York, USA; Emory University

**Keywords:** HIV, vaccine, immunogenicity

## Abstract

Recombinant soluble HIV-1 envelope glycoprotein (Env) SOSIP trimers are a design platform for inducing broadly neutralizing antibodies (bNAbs) by vaccination. To date, these and alternative designs of native-like trimers, given singly or in pairs, have not induced bNAbs in test animals such as rabbits or macaques. Here, we have evaluated whether trivalent and tetravalent combinations of SOSIP trimers from clades A, B, and C, delivered simultaneously or sequentially, induce better neutralizing antibody responses in rabbits than when given alone. None of the tested formulations led to the induction of bNAbs. We found that BG505 clade A trimers dominated the autologous NAb responses induced by combinations, which probably relates to the presence of immunodominant glycan holes on the BG505 trimer. Furthermore, autologous NAb responses to all individual trimers were reduced when they were delivered in combinations compared with when delivered alone, suggesting that immunogen interference had occurred. Finally, in a sequential regimen, a heterologous clade C trimer cross-boosted NAb responses that were primed by earlier immunizations with clade A and B trimers. Taken together, these findings should allow us to improve the design of immunization regimens based on native-like HIV-1 Env trimers.

**IMPORTANCE** A successful HIV-1 vaccine most probably requires a trimeric envelope glycoprotein (Env) component, as Env is the only viral protein on the surface of the virus and therefore the only target for neutralizing antibodies. Native-like Env trimers can induce strain-specific neutralizing antibodies but not yet broadly neutralizing antibodies. To try to broaden the antibody response, we immunized rabbits with soluble native-like Env trimers from three different clades using monovalent, multivalent, and sequential regimens. We found that the neutralizing antibody response against each immunogen was reduced when the immunogens were delivered in combination or sequentially compared to the monovalent regimen. In contrast, when the Env trimers from different clades were delivered sequentially, the neutralizing antibody response could be cross-boosted. Although the combination of native-like Env trimers from different clades did not induce broadly neutralizing antibodies, the results provide clues on how to use native-like trimers in vaccination experiments.

## INTRODUCTION

After more than 30 years of the HIV/AIDS epidemic, an effective vaccine is not yet available. One of the many barriers to overcome is the high diversity of viral strains. The isolation of a considerable number of broadly neutralizing antibodies (bNAbs) from infected individuals, i.e., antibodies that can neutralize multiple highly diverse HIV-1 isolates, has fueled hope that bNAbs can be elicited by appropriately designed vaccine regimens.

Soluble native-like envelope glycoprotein (Env) trimers that mimic the native HIV spike and that can induce neutralizing antibodies (NAbs) against relatively neutralization-resistant (tier 2) autologous viruses are a design platform for immunogens intended to induce bNAbs. Since the description of the prototype native-like trimer, BG505 SOSIP.664, many more SOSIP or derivative designs have been described (1–5). Trimers based on diverse genetic backgrounds, including from viruses of clades A, B, C, and G, are now available, and they generally induce autologous tier 2 NAb responses (4). The principal goal, to induce neutralization breadth, has not yet been achieved, but one strategy is to try to build on the narrow-specificity autologous responses (4).

Combination and sequential immunization strategies to broaden NAb responses have been analyzed by computational simulation. Thus, Wang et al. used a stochastic dynamic model to predict affinity maturation (6). When all types of immunogens were allowed simultaneously to encounter follicular dendritic cells (FDCs) for presentation to B cells, the model predicted that combination immunizations would not induce bNAbs because individual B cells would encounter different immunogens during the same and successive immunization rounds. The outcome would be conflicting selection forces during affinity maturation that would make it difficult to positively select specific, i.e., the most desired, B cells. The authors instead proposed an immunization scheme in which antigens are administered sequentially, a strategy predicted to favor the selection of the B cells that first interacted with the immunogens (6). In contrast, two other *in silico* studies favored the use of antigen combinations to increase breadth (7, 8). The authors considered the scenario in which each antigen contains both strain-specific and cross-reactive epitopes, which allowed them to model the specificity of the polyclonal response. Applied to a malaria vaccine, the model predicted that a combination of four antigenic variants would be the best way to drive broad neutralization responses (8). In another model, a B cell was allowed only one opportunity to interact with FDCs during each round of selection (7). A mixture of antigens and a range of conditions (number of antigens, concentrations, and mutational separation) were considered, leading to the conclusion that a mixture of three or four antigens would be nearly optimal for harnessing the conflicting forces that arise during the affinity maturation process required for bNAb development (7, 8).

Here, we have used several SOSIP trimers as sequentially or simultaneously delivered immunogens in strategies guided by the above-described theoretical considerations to seek neutralization breadth. We have previously reported the outcome of bivalent immunizations with the clade A BG505 and clade B B41 SOSIP.664 trimers (2, 9). Whether given sequentially or simultaneously to rabbits, these trimers both induced autologous NAb responses, but there was no increase in neutralization breadth. Subsequent boosting with trimers from a clade C virus, DU422, was also ineffectual in driving breadth, although immunizing with the clade B trimer and then its clade C counterpart did induce NAbs that could cross-neutralize the clade A BG505 virus (10). Further and more systematic studies are needed to determine whether multivalent combination and sequential immunizations might be more effective. We therefore used SOSIP.v4 and SOSIP.v5 trimer immunogens based on clade A (BG505), clade B (AMC008 and B41), and clade C (ZM197M) sequences (1–3, 9). The SOSIP.v4 and SOSIP.v5 designs incorporate additional stabilizing mutations that reduce the propensity of the trimer to undergo receptor-induced conformational changes, and they decrease the presentation of V3-directed and other nonneutralizing antibody (non-NAb) epitopes (3, 11). Here, we explored whether using these SOSIP trimers in combination (trivalent or tetravalent) or in a sequential formulation would increase the breadth of the NAb response. We observed that none of these strategies induced bNAbs, but we noticed that sequential immunization with trimers from different clades cross-boosted NAbs that neutralized clade-A BG505 virus.

## RESULTS

### Design of polyvalent and sequential trimer immunization regimens.

**FIG 1:**
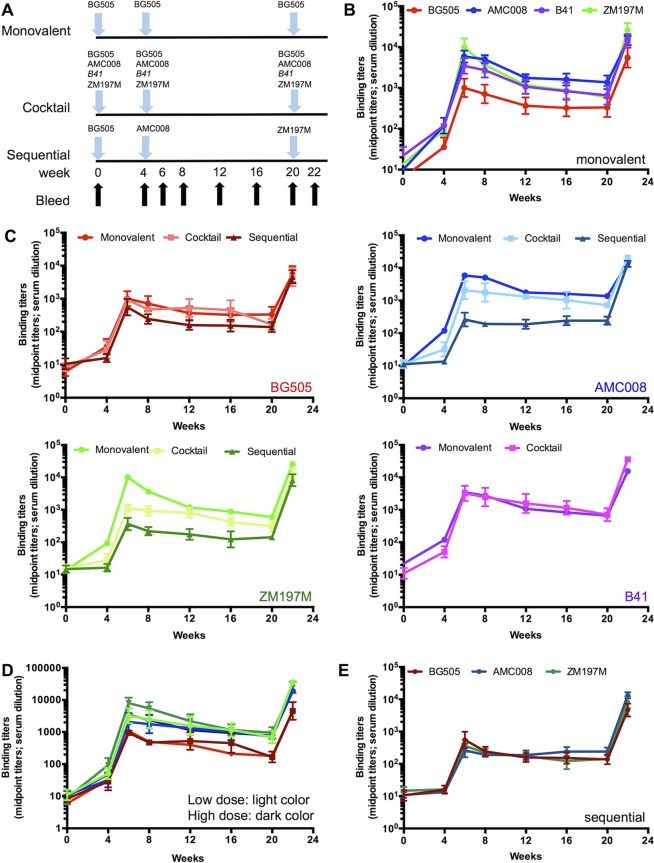
Monovalent and multivalent vaccine regimens and anti-trimer binding titers. (A) Schematic representation of the immunization schedule and regimens. Rabbits were immunized at weeks 0, 4, and 20 (blue arrows) with one or multiple trimers depending on the regimen. Serial bleeds were taken at weeks 0, 4, 6, 8, 12, 16, 20, and 22 (black arrows) for determination of anti-trimer and NAb titers. In the monovalent arms (groups 1 to 8; defined in Table S1 in the supplemental material), the rabbits received three doses of 22 μg of the same trimer (i.e., BG505, AMC008, B41, or ZM197M). In the combination arms (groups 9 to 13), the rabbits were given either a trivalent formulation of BG505, AMC008, and ZM197M trimers or a tetravalent formulation of the same trimers plus B41. In groups 10 and 12 the immunogen dose was 22 μg of each constituent trimer (high dose); in groups 9, 11, and 13 the total trimer dose was 22 μg (low dose). In the sequential immunization arm (group 14), the rabbits received 22 μg of a different trimer at each immunization (first, BG505; second, AMC008; third, ZM197M). (B) Anti-trimer binding Ab titers induced by monovalent trimers. The midpoint binding titer (EC_50_) over time is shown for each immunogen. (C) Anti-trimer binding Ab titers induced by each trimer in the monovalent, combination, or sequential immunization groups. The midpoint binding titers (EC_50_) induced by the different regimens are displayed for each trimer immunogen: BG505 in different shades of red, upper left; AMC008 in different shades of blue, upper right; ZM197M in different shades of green, lower left; B41 in purple and magenta, lower right. (D) Midpoint anti-trimer titers (EC_50_) induced by each trimer in the low (light color)- and high (dark color)-dose combination groups. (E) Anti-trimer binding Ab titers induced by each trimer in the sequential formulation.

We compared the immunogenicity of three different immunization regimens in groups of five rabbits. Single (monovalent) PGT145 affinity-purified SOSIP.v4 or SOSIP.v5 trimers were compared with combinations of trimers given either simultaneously or sequentially (Fig. 1A). The 40 animals in monovalent groups 1 to 8 (see Table S1 in the supplemental material) received three immunizations with 22 μg of trimer (details in Table S1). These monovalent immunogen groups serve as the comparators between studies for the more complex regimens and have been previously described (3, 11). They include historic and contemporaneous control groups (see rabbit study number in Table S1).

In total, 25 animals (groups 9 to 13) received three immunizations with a trivalent (groups 9 and 10) or tetravalent (groups 11 to 13) trimer combination. In the trivalent groups, three immunizations of BG505, AMC008, and ZM197M SOSIP.v4 trimers were given in either a low dose (7.3 μg of each, i.e., 22 μg in total; group 9) or a high dose (22 μg of each, i.e., 66 μg in total; group 10). The tetravalent groups received three immunizations with BG505, AMC008, B41, and ZM197M trimers, again either at a low dose (5.5 μg of each, i.e., 22 μg in total; groups 11 and 13) or a high dose (22 μg of each, i.e., 88 μg in total; group 12). In the tetravalent study, the rabbits in group 11 received SOSIP.v4 trimers (3), while those in group 13 were given the SOSIP.v5 variants (11).

Finally, the group 14 rabbits were given sequential immunizations with 22 μg of SOSIP.v5 trimers in the following order: BG505 at week 0, AMC008 at week 4, and ZM197M at week 20. This sequence was chosen based on the observation that the BG505 trimer is more immunogenic (for the autologous NAb response) than AMC008 and ZM197M (3, 11) and because it has a less dense glycan shield than the other two (i.e., BG505 contains 28 potential N-linked glycosylation sites [PNGS], whereas AMC008 and ZM197M have 31 and 34 PNGS, respectively) (Table 1) (11, 12). Thus, the AMC008 trimer contains a less dense glycan shield than the ZM197M trimer, which has a comparatively dense and more complete glycan shield (Table 1). The overarching rationale was that a gradual increase in the density of the glycan shield might drive the maturation of cross-reactive NAbs (see Discussion).

**TABLE 1:**
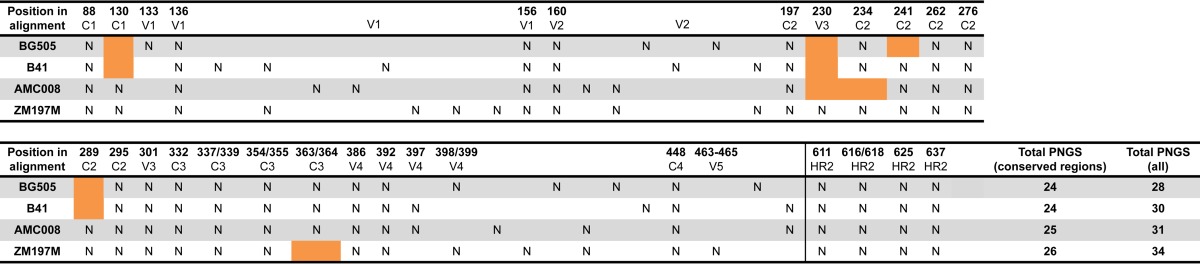
Summary of potential N-linked glycosylation sites in BG505, AMC008, B41, and ZM197M immunogens^*a*^

a PNGS are numbered according to their position in the sequence aligned to that of HXB2. Orange boxes indicate positions in conserved regions that frequently harbor a PNGS (≥50% frequency across HIV strains and types) but where a PNGS is absent from BG505, AMC008, B41, or ZM197M.

### Autologous anti-trimer binding antibody responses.

**FIG 2:**
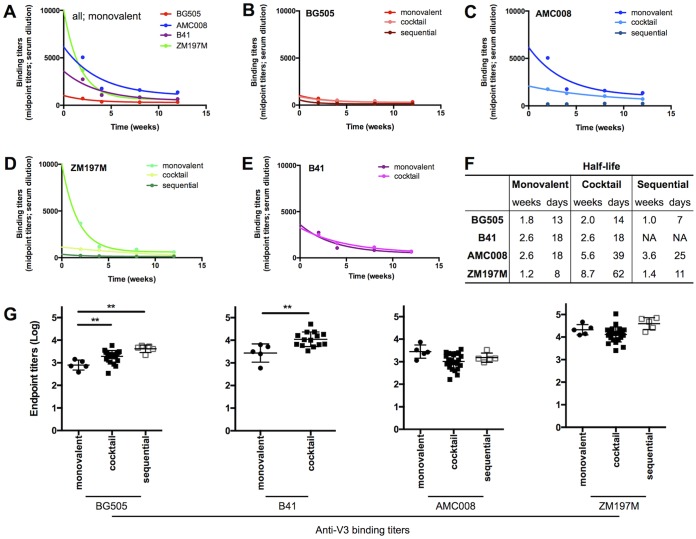
Decay rates of anti-trimer binding titers and V3 peptide binding titers in monovalent, combination, and sequential regimens. (A to E) The decay curves of anti-trimer binding Ab titers (EC_50_) after the second vaccination (i.e., between weeks 6 and 20) with a single (monovalent) or multiple trimer(s) are shown. In each panel, the median anti-trimer Ab titers (EC_50_s) and the nonlinear regression curves of one-phase decay unconstrained equations are displayed. (A) Decay curves of anti-trimer binding Ab titers in monovalent regimens. (B to E) Comparison of decay curves of anti-trimer binding Ab titers in monovalent, combination, and sequential regimens for BG505 (B), AMC008 (C), ZM197M (D), and B41 (E). (F) Summary of half-lives (in weeks and days) of the Ab binding titers between weeks 6 and 20, calculated for each immunogen with the previously mentioned one-phase unconstrained equation (see Materials and Methods). (G) The midpoint V3 peptide binding Ab titers (EC_50_) were calculated by ELISA and plotted.

The time course of binding Ab responses against the immunogen trimers, measured as median titers across each group, was assessed by capture enzyme-linked immunosorbent assay (ELISA). In the animals immunized with single trimers, the autologous anti-trimer ELISA titers increased sharply between weeks 4 and 6 and then again between weeks 20 and 22, i.e., after the second and third immunizations, but declined after each of these boosts (Fig. 1B). The half-lives (*t*_1/2_) of the titer decays between weeks 6 and 20 were significantly shorter for ZM197M and BG505 (8 and 13 days) than for AMC008 or B41 (both 18 days; *P* value of 0.0041) (Fig. 2A to F). The general profile of autologous binding Ab titers over time against the AMC008, B41, and ZM197M trimers were similar, while those induced by BG505 trimers were lower, although not to a statistically significant extent (*P* value of 0.0556) (Fig. 1B).

The autologous anti-trimer ELISA titers varied between different immunization regimens. The binding Ab titers induced by BG505 or B41 trimers were similar over time whether they were delivered as individual immunogens or in multivalent combinations, while the responses to AMC008 and ZM197M trimers were slightly weaker for combination regimens than for the monovalent ones, but not to a statistically significant extent (Fig. 1C). The quantity of trimers used for immunizations (low- versus high-dose formulation) did not influence the resulting autologous anti-trimer titers (Fig. 1D). Sequential immunizations generally resulted in lower anti-trimer titers, but the maximum titer depended on when a particular trimer was given (Fig. 1C). Thus, at week 6, the responses to BG505, the first in the sequential regimen, were similar to those in the monovalent BG505 trimer group (compare Fig. 1C and A). They remained somewhat lower in the sequential than in the monovalent group until week 22, suggesting that the BG505 trimer-primed responses were cross-boosted efficiently by the later AMC008 and ZM197M immunizations. By week 22 in the sequential regimen, the anti-BG505, AMC008, and ZM197M trimer titers were similar to those in the corresponding monovalent groups (Fig. 1E). Since a different outcome was seen when NAb responses at week 22 were compared (see below), the anti-trimer binding Abs induced in the sequential immunization groups may substantially reflect the priming and cross-boosting of mostly nonneutralizing antibodies (non-NAbs).

As anti-V3 non-NAbs are known to contribute strongly to the anti-trimer ELISA titers, we measured endpoint titers against autologous V3 peptides at week 22 (3). The anti-V3 titers for BG505 and B41 were significantly higher in the combination than in the monovalent groups (*P* value of 0.0041 for BG505 and *P* value of 0.0033 for B41). Thus, when given in combination, these two Env trimers cross-boosted anti-V3 non-NAbs (Fig. 2G). However, this was not the case for AMC008 and ZM197M, as these anti-V3 titers were similar in the monovalent and combination groups, suggesting that other non-NAb responses were cross-boosted (Fig. 2G). In the sequential immunization regimen, the anti-BG505 V3 titer was higher than that in the monovalent BG505 group at week 22 (*P* value of 0.0079) (Fig. 2G), but the AMC008 and ZM197M V3 titers were similar in the two immunization groups (Fig. 2G). Overall, this indicates that while the V3 of BG505 and B41 might be more antigenic after immunization, V3 responses against AMC008 and ZM197M are more difficult to cross-boost.

### NAbs against autologous viruses.

**FIG 3:**
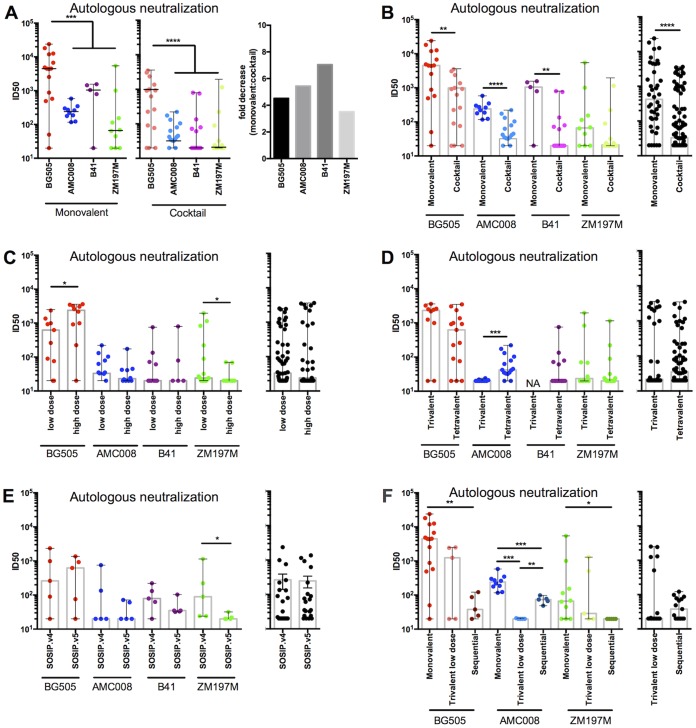
Autologous NAbs induced by monovalent and multivalent regimens. NAb titers at week 22 were assessed in the TZM-bl assay (9, 10). Individual ID_50_ values are reported in Table S1 in the supplemental material. The bars show the median midpoint neutralization titers (ID_50_) and ranges. (A) Autologous NAb titers for the monovalent (left) and combination (middle) groups, with BG505 in red, AMC008 in blue, B41 in purple, and ZM197M in green. The bar chart on the right shows the fold decrease in autologous NAb median ID_50_ values between the monovalent and combination formulation groups. (B to F, left) Autologous NAb titers (ID_50_ values) for each trimer/virus pair. (Right) Pooled autologous NAb titers for all of the trimers and the corresponding viruses. The comparisons of autologous NAb titers are the following: monovalent versus combination regimens (B); low- versus high-dose combinations (C); trivalent versus tetravalent combinations (D); SOSIP.v4 versus SOSIP.v5 trimers in combination (E); monovalent versus low-dose trivalent versus sequential formulations (F).

When autologous NAb responses were measured 2 weeks after the last immunization (i.e., at week 22), the immunogenicity differed among the trimers, consistent with previous reports (3, 10, 11). Thus, the BG505 trimers induced ∼10-fold higher autologous NAb titers as a monovalent immunogen than did AMC008, B41, or ZM197M (*P* value of 0.0003) and ∼30-fold higher titers when given as part of a combination than those of other trimers in combination pools (*P* < 0.0001) (Fig. 3A, left).

Sera from animals immunized with a single trimer consistently neutralized the corresponding autologous tier 2 virus more potently than sera from the combination groups (Fig. 3B). Thus, the BG505 NAb titer for the monovalent group was 4.5-fold higher (*P* value of 0.0083) than that for the combination groups. One explanation is interference by the other trimers in the mixture, and another is a dose reduction effect in some of the combinations (see below). Similar findings applied to the other trimers (AMC008, ∼7.5-fold lower for combination groups than for monovalent, *P* value of 0.0081; B41, ∼50-fold lower, *P* value of <0.0001; ZM197M, ∼3-fold lower, not significant [NS]; all four viruses combined, ∼6-fold lower, *P* value of <0.0001).

To assess whether dose variation had any influence, we next compared the monovalent groups with only the high-dose combination groups, as the 22-μg dose was the same in each case. The BG505 NAb titers were now ∼2-fold higher in the monovalent group than in the high-dose combination group (NS), but greater differences were found for the other trimers (AMC008, ∼6-fold difference, *P* value of 0.0045; B41, ∼52-fold difference, *P* value of <0.0001; ZM197M, ∼3-fold difference, NS). These differences were not seen in the anti-trimer binding antibody analyses described above, implying that the latter assay outcomes were skewed by the presence of substantial amounts of non-NAbs (Fig. 1C, week 22). Overall, the autologous NAb responses are reduced when trimers are used in multivalent formulations (Fig. 3B). The extent of this reduction for combination compared with the monovalent formulation was greater for the AMC008 and B41 trimers than for BG505 (Fig. 3B). It is, therefore, possible that the BG505 trimer dominates in the induction of NAbs at the expense of those elicited by its AMC008 and B41 counterparts when they are codelivered.

We then compared the low- and high-dose multivalent groups. The BG505 trimers induced ∼4-fold higher autologous NAb titers at the high dose than the low dose (*P* value of 0.018) (Fig. 3C). In contrast, for ZM197M the stronger responses were seen in the low-dose groups (2 of 10 animals had NAb titers of >40 in the high-dose groups but 5 of 10 at the lower dose; *P* value of 0.0360) (Fig. 3C). Hence, immunogen interference may be more likely than a dose reduction effect to account for any reduced immunogenicity of the ZM197M trimer in combinations. When we compared the autologous NAb responses in the tetravalent and trivalent groups, neutralization of AMC008 was consistently moderate in the tetravalent group (8 of 10 versus 0 of 10 sera with NAb titers of >40, respectively; *P* value of 0.0002) (Fig. 3D). One possibility is that the presence of additional clade B trimers (i.e., B41) in the tetravalent combination induced cross-reactive, low-titer NAbs against AMC008. The autologous NAb titers for BG505 and ZM197M (B41 was only given in the trivalent combination) did not differ between the tetravalent and trivalent formulations.

We next assessed whether trimer design SOSIP.v4 or SOSIP.v5 influenced the induction of autologous NAbs in the low-dose tetravalent combination groups. While we did not see a difference in BG505, AMC008, and B41 NAb responses, we observed that the ZM197M SOSIP.v4 trimers induced stronger, although still weak, autologous NAb responses than the SOSIP.v5 variant (*P* value of 0.0317) (Fig. 3E).

At week 22 in the sequential immunizations, autologous NAb responses against BG505 and AMC008 were weak, and there was no neutralization of ZM197M (Fig. 3D). In general, autologous NAb responses appear to require at least two immunizations with the same trimer (10), which reduces the comparability of the sequential regimen with the other groups. The cumulative immunogen dose administered in the sequential regimen is closest to the low-dose trivalent group, where the rabbits received the same amount (22 μg) of all three trimers but split between three immunizations (Fig. 3F). In the sequential group, 4 of 5 rabbits induced NAbs against BG505 but at titers 4.5-fold lower than those in the monovalent group (*P* value of 0.0039) and 3.2-fold lower than those in the low-dose trivalent combination groups (NS). For AMC008, the corresponding NAb titer reduction compared with the monovalent regimen was ∼3-fold (*P* value of 0.0007), but the NAb titer was ∼4-fold higher than that in the low-dose trivalent regimen (*P* value of 0.0079). For ZM197M, 2 of 5 rabbits in the low-dose trivalent group had measurable autologous NAbs (titers of >40), but 0 of 5 in the sequential group did so (NS), and for the monovalent group the corresponding frequency was 7 out of 10 (Fig. 3F). Thus, overall the sequential regimen was inferior to the combination regimen at inducing autologous antibodies.

**FIG 4:**
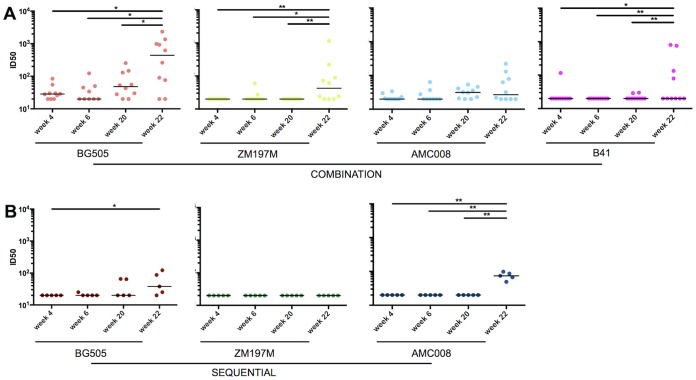
Autologous NAbs induced by combination and sequential regimens over time. NAb titers (ID_50_) at weeks 4, 6, 20, and 22 were assessed in the TZM-bl assay. Individual ID_50_ values are reported in Table 2. Median midpoint neutralization titers are shown. (A) Autologous NAb titers in the combination regimen induced by BG505, ZM197M, AMC008, and B41. (B) Autologous NAb titers in the sequential regimen.

In the sequential immunization group, NAbs against BG505 and AMC008 appeared only after the third immunization, i.e., with the ZM197M trimer. The explanation could be that this heterologous trimer cross-boosted NAb responses to AMC008 and BG505 that were primed by the first two immunizations with the BG505 and AMC008 trimers, respectively (Fig. 4A and B and Table 2). This observation is consistent with the low-level cross-neutralization of both BG505 and AMC008 seen in the monovalent ZM197M trimer group (Table S1).

**TABLE 2:**
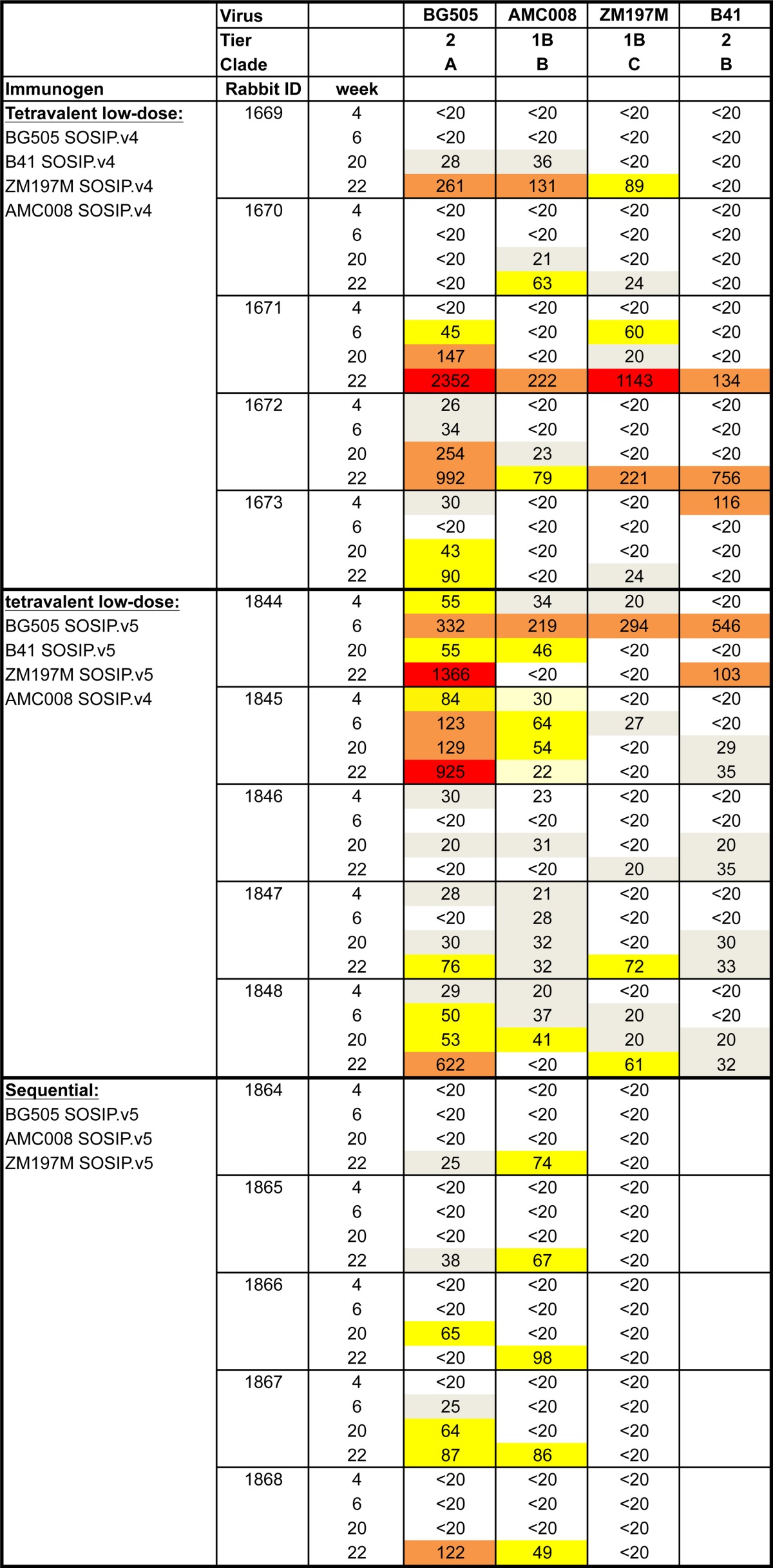
Autologous neutralizing antibody titers induced by each immunogen over time^*a*^

a The TZM-bl assay was performed at the Academic Medical Center (AMC). ID_50_ values are shown and colored according to their magnitude. White, ID_50_ < 20; gray, 20 < ID_50_ < 40; yellow, 40 < ID_50_ < 100; orange, 100 < ID_50_ < 1,000; red, ID_50_ > 1,000.

### NAbs against heterologous viruses.

**FIG 5:**
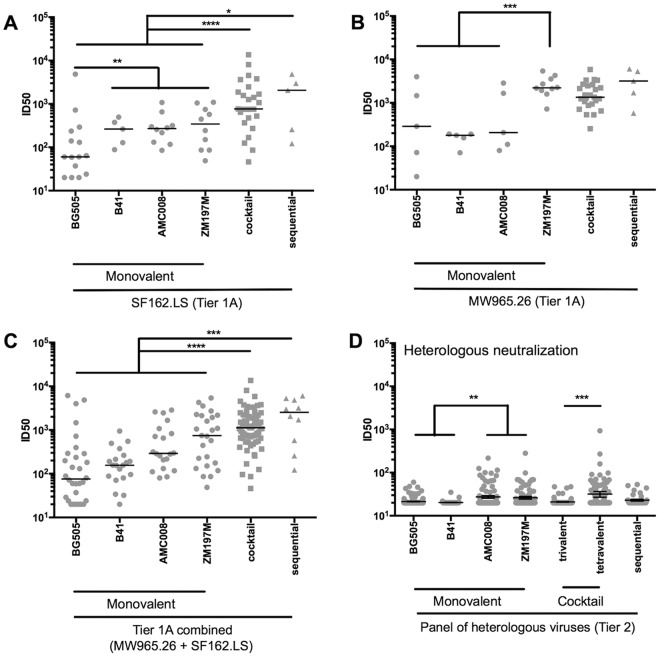
Heterologous NAbs induced by monovalent and multivalent regimens. The midpoint NAb titers (ID_50_) against tier 1A viruses SF162 (A) and MW965.26 (B) as determined using TZM-bl assay are plotted, while both data sets are combined in panel C. (D) Heterologous NAb titers (ID_50_) against 16 heterologous tier 2 viruses are shown. Individual ID_50_ values are reported in Table S1 in the supplemental material.

To assess neutralization breadth, we tested sera from every rabbit at week 22, first against two heterologous tier 1A (SF162.LS, clade B, and MW965.26, clade C) and then 16 tier 2 Env-pseudotyped viruses (Table S1). The BG505 SOSIP.v4.1 and SOSIP.v5.2 trimers induced a weak NAb response against SF162.LS (median titer, 60), consistent with previous reports on these immunogens (Fig. 5A) (3, 11). The SF162.LS NAb response was ∼4-fold higher in rabbits given AMC008, B41, or ZM197M trimers (*P* value of 0.0051 for the pooled data set compared with BG505). In the sequential and combination immunizations, the SF162.LS NAb titers were ∼3-fold greater for the combination groups than those for the pooled monovalent groups (*P* < 0.0001) and ∼8-fold higher than those for the sequential groups (*P* value of 0.0394). NAb titers against MW965.26 were moderate for monovalent BG505-, AMC008-, and B41-immunized groups (median titer, 100 to 300) (Fig. 5B) but significantly higher in animals given the clade C-matched ZM197M trimers (median titer, 2,000; *P* value of 0.0009 compared with the other clades pooled) (Fig. 5B). The MW965.26 NAb responses in the combination and sequential regimen groups were similar to those in the monovalent ZM197M trimer group. Overall, pooling tier 1A NAb data for both viruses showed that the combination and sequential regimens induced significantly higher titers than the monovalent regimens (combination, ∼5-fold higher, *P* value of <0.0001; sequential, ∼12-fold higher, *P* value of 0.0006) (Fig. 5C).

The monovalent trimer formulations did not induce consistent heterologous tier 2 NAb responses, as described previously (3, 10, 11, 13). However, sera from some rabbits immunized with AMC008 and ZM197M trimers did neutralize a few tier 2 viruses with moderate titers (>100) (Fig. 5D and Table S1).

Autologous tier 2 NAbs induced in rabbits and macaques generally target holes in the glycan shield (10, 12, 14). Here, we hypothesized that density of glycans in the glycan shield of the immunogen trimers would correlate with the induction of heterologous tier 2 NAbs. The underlying argument is that immunodominant hole-directed autologous NAbs might interfere with the induction of cross-reactive NAbs against less immunogenic sites (10, 12). The BG505 and B41 trimers contain a less dense glycan shield than AMC008 and ZM197M, involving PNGS at 28 (BG505) and 30 (B41) asparagines, whereas the AMC008 trimer contains 32 PNGS (Table 1). In contrast, the ZM197M trimer, based on our analysis of its amino acid sequence, has a very dense glycan shield, consisting of 34 PNGS (Table 1). We therefore compared the outcomes of immunizing with trimers with less dense glycan shields (i.e., BG505 and B41) and ones with denser glycan shields (i.e., AMC008 and ZM197M, respectively). Sera from rabbits immunized with the AMC008 and ZM197M trimers neutralized (titers of >40) significantly more heterologous tier 2 viruses than their less shielded counterparts. Specifically, the number of heterologous viruses hit was 8 for AMC008 and 8 for ZM197M versus 2 for BG505 and 0 for B41 (*P* value of <0.0001 for AMC008 and ZM197M compared to BG505 and B41) (Fig. 5D).

The trivalent, tetravalent, and sequential trimer regimens did not induce more potent or frequent heterologous tier 2 NAbs than the monovalent groups (Fig. 5D and Table S1). However, the heterologous NAb responses were more frequent and showed higher titers in the tetravalent than the trivalent group (*P* < 0.0001) (Fig. 5D). The B41 trimer was the one present only in the tetravalent combinations, so it is possible that it contributed to the increased breadth despite its inability to elicit heterologous tier 2 NAbs as a monovalent immunogen.

Overall, the SOSIP trimers differed in their capacity to induce heterologous NAbs, which may be related to the completeness of their glycan shields. However, simply mixing trimers from different clades is not sufficient to induce neutralization breadth.

## DISCUSSION

Sequential and cocktail immunization strategies have previously been explored for HIV-1 Env immunogens using DNA vaccines, recombinant gp120, or uncleaved gp140 immunogens (15–20). Generally, these studies showed that cocktail and sequential immunization regimens induced higher NAb responses against easy-to-neutralize (tier 1) viruses than monovalent vaccines, and our findings are consistent with this (Fig. 5A to C). However, these earlier immunogens did not consistently elicit NAb responses against difficult-to-neutralize (tier 2) viruses that are representative for the majority of circulating primary HIV-1 isolates. The ability of native-like trimers to induce tier 2 NAbs consistently prompted us to assess the capacity of native-like trimers from clades A, B, and C to generate NAbs when administered to rabbits in combination or sequentially. Our principal conclusion is that neither strategy is sufficient to elicit bNAbs. However, several secondary conclusions may guide improvements to trimer-based bNAb induction programs.

First, our results suggest that some trimers, particularly BG505, are immunodominant when used in combination with others. This observation is consistent with the strong immunogenicity of the BG505 trimer when used alone (3, 11, 13). Thus, even though all four trimers in a tetravalent combination elicited autologous NAbs, the highest titers against BG505 and AMC008 failed to elicit autologous NAbs in a trivalent combination. Moreover, the BG505 trimer was more prone than the other three trimers to interfere with the other components of the combinations (see below). One plausible explanation is that the presence of the large immunodominant glycan hole around residues 241 and 289 skews the response toward the BG505 trimer rather than generating a more balanced set of NAbs to the full combination (10, 12). In this context, we noted that the BG505 and B41 trimers, which lack two and three relatively conserved PNGS, respectively, induce strong autologous tier 2 NAbs but with few if any signs of cross-neutralizing antibodies. The AMC008 and ZM197M trimers, which lack only one or no conserved PNGS, induce lower titers of autologous NAbs but raise heterologous tier 2 NAbs somewhat more frequently (although still sporadically). It seems possible that the presence of a dense glycan shield on a trimer might help to drive the NAb response toward less immunogenic but more conserved epitopes. Filling holes by introducing glycans into trimers such as BG505 may be a path to explore in the quest for cross-reactive NAbs.

We also found evidence for immune interference when trimers were given in combinations, with the effect being a disproportional reduction in the induction of autologous NAbs by some components. In short, autologous NAb titers were overall lower in combination or sequential immunizations than in monovalent regimens. Models of how GCs respond to immunogen combinations suggest that when individual B cells encounter different antigens, the conflicting selection forces during affinity maturation favor B cell apoptosis, a scenario that might be consistent with our findings (6). However, the same models also suggest that sequential immunogen regimens should drive broader NAb responses, since B cells that target relatively conserved epitopes would be activated by each immunogen boost. However, we did not observe this. When bNAbs emerge during HIV-1 infection after multiple rounds of affinity maturation, the antigens involved are usually more closely related than the sequence-diverse trimers from clades A, B, and C we used in this study. The scenario found during infection might lead to the preferential activation of B cells recognizing shared epitopes and therefore fewer conflicting signals to the B cells. Several groups have studied Env evolution in HIV-1-infected individuals that developed bNAbs (21–25). Hence, sequential and combination regimens with closely related Env sequences from longitudinal samples from individual patients become feasible and should be tested.

Our third observation is that the B cell responses to trimers in combination are independent of one another, such that multiple autologous NAbs, but not cross-reactive ones, emerge. However, autologous NAbs against all of the immunogen trimers were seen only for the tetravalent combination and not the trivalent one. An explanation may be that the presence of two clade B trimers (B41 and AMC008) in the tetravalent regimen strengthened the NAb response against AMC008. Again, the use of more closely related trimers might be beneficial for driving some degree of neutralization breadth. Trimers derived from evolving Env sequences in the same HIV-1-infected individual might be particularly worth studying in this regard.

We also found that the binding titers in the sequential and combination groups at week 22 all reached a similar level, which must have involved cross-boosting. However, since this breadth is not in line with the NAb titers, we can conclude that the cross-boosted responses mostly comprise non-NAbs, which are particularly easy to induce and therefore to cross-boost, as previously reported. Further research should assess whether diminishing the non-NAb responses would increase the breadth of the NAb responses.

Finally, in the sequential immunogen group we found that clade C trimers cross-boosted NAb responses active against the clade A and B viruses that had been primed by the earlier clade A and B trimers. For this to happen, some common antigenic determinants relevant to neutralization must be shared on these sequence-divergent trimers; we note that their antigenicities and structures may differ much less than their primary sequences. In principle, such observations could be exploited in the design of future immunization schemes for the elicitation of more broadly active NAbs.

When testing cocktail regimens, we found that tetravalent combinations based on SOSIP.v4 or SOSIP.v5 induced comparable autologous NAb responses, suggesting that the SOSIP version and thermostability are not a major determinant for the outcome of our experiments. This is consistent with the observation that SOSIP version and thermostability also were not a determining factor for the induction of autologous NAb responses in immunizations with monovalent vaccines (3, 11). The stability of the trimer appears to play a less dominant role than the viral isolate from which the trimer is derived, and as a consequence, the strength of the NAb response differs between trimers from different viral isolates more so than between different SOSIP trimers from the same viral strain (3, 11, 13).

In conclusion, the induction of bNAbs requires more than the use of randomly chosen clade-divergent trimers delivered sequentially or in trivalent/tetravalent combinations. Nonetheless, we found some clues to guide the design of superior strategies based on the use of more closely related trimers, such as those derived from patient-derived lineages, which could be tested experimentally.

## MATERIALS AND METHODS

### Immunogens.

Native-like trimers based on BG505 (clade A, tier 2), AMC008 and B41 (both clade B, tier 1B and tier 2, respectively), and ZM197M (clade C) were used as immunogens (1–3). Some trimers were based on the SOSIP.v4 design, and others were based on SOSIP.v5 (3, 11) (see Table S1 in the supplemental material). D7324-tagged variants of the same trimers were used in anti-trimer capture ELISAs as summarized below and described elsewhere (3).

### Immunizations.

Rabbit immunizations were performed under contract at Covance Research Products, Inc. (Denver, PA, USA), under permits with approval numbers C0022-15, C0045-15, C0119-15, and C0120-15. The immunization and bleeding schedules are summarized in Fig. 1A. In summary, in the monovalent and sequential groups, female New Zealand White rabbits (*n* = 5 per group) were immunized at weeks 0, 4, and 20 with 22 μg of trimer (based on peptidic mass) formulated in Iscomatrix adjuvant, as published previously (3, 10, 11, 13). In the combination groups, the animals received either 22 μg of each trimer (high dose) or a total trimer amount of 22 μg (low dose) with the same adjuvant.

### Neutralization assays.

Autologous and heterologous NAbs were measured in the TZM-bl cell neutralization assay using sera from week 22, as previously described (13). Viruses of defined tier classifications were used (26).

### Anti-trimer binding antibody ELISA.

Titers of trimer binding Abs were assessed using the D7324-capture ELISA described elsewhere (9, 13). Rabbit sera from various time points (Fig. 1A) were titrated from a starting dilution of 1:100, and the midpoint titers (50% effective concentration [EC_50_]) were determined.

### V3 peptide ELISA.

Antibodies reactive with V3 peptides were quantified in animals from groups 2, 4, 5, 7, and 9 to 14 as previously described (3). The V3 peptides were identical to the immunogen and were based on the BG505, B41, AMC008, or ZM197M sequence. The peptides were cyclized by a disulfide bond between residues 1 and 35 and included the A316W mutation that is present in SOSIP.v4 and SOSIP.v5 trimers.

### Statistics and half-life calculations.

Differences between rabbit groups in anti-trimer and autologous NAb titers, as well as measurements of neutralization breadth, were assessed by two-tailed Mann-Whitney U test. The half-life (*t*_1/2_) of the binding Ab responses after the second immunization, i.e., between weeks 6 and 20, were calculated with GraphPad Prism using the one-phase unconstrained equation *Y* = (*Y*_0_ − plateau) × exp(−*K* × *X*) + plateau. *Y*_0_ is the binding titer when time is 0. Plateau is the binding titer when time is infinite. *K* is the constant rate expressed in reciprocal of days (days^−1^). The half-life is computed as ln(2)/*K* and expressed in days.

## Supplementary Material

Supplemental material
