# Supplementary material for: Immunogenicity in Rabbits of HIV-1 SOSIP Trimers from Clades A, B, and C, Given Individually, Sequentially, or in Combination
Source: J Virol. 2018 Mar 28;92(8):e01957-17. doi: 10.1128/JVI.01957-17 (PMC5874403; doi:10.1128/JVI.01957-17)
Supplement: Supplemental material [file supp_92_8_e01957-17__index.html]

Immunogenicity in Rabbits of HIV-1 SOSIP Trimers from Clades A, B, and C, Given Individually, Sequentially, or in Combination — Supplemental material 

# Immunogenicity in Rabbits of HIV-1 SOSIP Trimers from Clades A, B, and C, Given Individually, Sequentially, or in Combination

## Supplemental material

- Supplemental file 1 -

  Table S1 (Comparison of the immunogenicity of three different immunization regimens in groups of rabbits immunized with clade A, clade B, and clade C immunogens.)

  XLSX, 55K
- Supplemental file 2 -

  Legend to Table S1

  PDF, 31K
