# Supplementary material for: Immunogenicity in Rabbits of HIV-1 SOSIP Trimers from Clades A, B, and C, Given Individually, Sequentially, or in Combination
Source: J Virol. 2018 Mar 28;92(8):e01957-17. doi: 10.1128/JVI.01957-17 (PMC5874403; doi:10.1128/JVI.01957-17)
Supplement: Supplemental material [file JVI.01957-17_zjv007183439s2.pdf]

Table S1. Midpoint neutralization titers (ID_{50}) against a panel of Env pseudotyped viruses from rabbits immunized with clade A, clade B and clade C immunogens in monovalent, combination and sequential regimens. The TZM-bl assay was performed at the Academic Medical Center (AMC) or at the Duke University Medical Center (DUMC). ID_{50} values are shown and colored according to their magnitude. ID_{50} <20 in white; 20<ID_{50}<40 in grey; 40<ID_{50}<100 in yellow; 100<ID_{50}<1000 in orange and ID_{50}>1000 in red.
